# Implementation of hip replacement surgery recommendations: a qualitative study of orthopaedic surgeons’ perspectives

**DOI:** 10.1186/s12891-025-09334-z

**Published:** 2025-12-08

**Authors:** Rachael Powell, Amy Davies, Tony Coffey, Vikki Wylde, Helen Hickey, Hiren Divecha, Tim N. Board

**Affiliations:** 1 Manchester Centre for Health Psychology, Division of Psychology and Mental Health, School of Health Sciences, Faculty of Biology, Medicine & Health, University of Manchester, Manchester, UK; 2 Division of Nursing, Midwifery and Social Work, School of Health Sciences, Faculty of Biology, Medicine & Health, University of Manchester, Manchester, UK; 3 Liverpool Clinical Trials Centre, University of Liverpool, Liverpool, UK; 4 Musculoskeletal Research Unit, Bristol Medical School, University of Bristol, Bristol, UK; 5 NIHR Bristol Biomedical Research Centre, University Hospitals Bristol and Weston NHS Foundation Trust and University of Bristol, Bristol, UK; 6 The Centre for Hip Surgery, Wrightington, Wigan and Leigh Teaching Hospitals NHS Foundation Trust, Wigan, UK; 7 Division of Musculoskeletal and Dermatological Science, School of Biological Sciences, Faculty of Biology, Medicine & Health, University of Manchester, Manchester, UK

**Keywords:** Total hip arthroplasty, Hip replacement, Surgery, Implementation, Qualitative, Implant fixation, Hybrid, Cemented, Feasibility study

## Abstract

**Background:**

Total hip replacement is a common surgical procedure in which the ‘ball’ and ‘socket’ components of the hip joint are replaced with implants. The HipHOP study (Hip arthroplasty with Hybrid Or cemented implants: Patient reported outcomes) evaluated the feasibility of conducting a randomised controlled trial of two implant types. Qualitative research was embedded within the HipHOP study and aimed to understand factors which might affect the implementation of a future trial’s findings.

**Methods:**

Semi-structured qualitative interviews were conducted by telephone with sixteen orthopaedic surgeons. Purposive sampling ensured inclusion of surgeons who did and did not agree to their patients being included in the feasibility trial at two hospital sites, and also surgeons at a further two sites which were not involved in the feasibility trial. An inductive, thematic analysis was conducted, structured using the Framework approach. Findings were mapped to the Theoretical Domains Framework (TDF) and the Consolidated Framework for Implementation Research (CFIR).

**Results:**

To facilitate implementation of trial findings, it seemed important that advice be based on good quality research evidence showing clear benefits to the recommended approach and be supported by trusted professional organisations. Participants also considered the influence of surgeons’ personal surgical results, recognising that outcomes may be affected by an individual surgeon’s training and experience with an implant type. The importance of enabling surgeons to gain necessary skills was highlighted. The TDF seemed to be particularly valuable in understanding factors underlying implementation behaviour in this context, with the CFIR showing potential to contribute to understanding within some areas.

**Conclusions:**

For a trial’s findings to be influential in changing practice, it seems that: the trial needs to be well-designed and provide clear, strong findings; surgeons need to believe that changing practice will improve their personal outcomes; and suitable training opportunities need to be provided where surgeons lack experience using a recommended implant type.

**Trial registration:**

Registered 19/02/2021, ISRCTN11097021.

**Supplementary Information:**

The online version contains supplementary material available at 10.1186/s12891-025-09334-z.

## Introduction

Total hip replacement (THR) is a common elective surgical procedure conducted to reduce pain and functional limitations. Over 100,000 THR operations are carried out annually in the UK [1, 2]. A THR involves replacing both the ‘socket’ and ‘ball’ of the hip joint. There are many different implants that can be used. Two implant types, categorised by the method used to fix the implant to bone, are fully cemented (socket and stem are fixed with cement) and hybrid (cemented stem and uncemented socket). Both implant types are commonly used, but there is insufficient evidence establishing which leads to optimum outcomes. The HipHOP (Hip arthroplasty with Hybrid Or cemented implants: Patient reported outcomes) feasibility study aimed to evaluate the feasibility and acceptability of a randomised controlled trial (RCT) to investigate the clinical and cost-effectiveness of cemented versus hybrid THR implants [3]. Reverse hybrid implants (where the femur is uncemented but socket is cemented) were not included in the HipHOP feasibility study because these are less commonly performed in practice by surgeons than cemented and hybrid implants. There is also an ongoing large RCT comparing cemented, uncemented and hybrid implants in patients aged less than 70 years [4]. Having good quality evidence indicating which implant type is the most clinically and cost-effective could have considerable impact on patient outcomes and health service costs. However, impact requires that evidence-based guidelines are implemented in practice.

It is well established that research evidence is not always implemented into clinical practice [5]. This has been observed in orthopaedic surgery, resulting in the recommendation for research to understand surgeons’ reasons for preferring approaches which are not best supported by research evidence [6]. Medical Research Council guidance advises that implementation issues be considered from early research stages to raise the likelihood that an intervention can be successfully implemented into healthcare [7]. Implementing recommendations requires changes in human behaviour, and it is important to understand what the determinants of a behaviour are – what “needs to change” to enable a behaviour – to effectively support behaviour change ([8], p57).

Relevant theory should be used when considering implementation in order to have a coherent understanding of the necessary processes for change [9]. Two theory-based frameworks developed to draw together theoretical constructs relevant to intervention implementation are the Theoretical Domains Framework (TDF) [9, 10] and the Consolidated Framework for Implementation Research (CFIR) [11]. The TDF contains 14 domains, including ‘Knowledge’, ‘Skills’, ‘Social/professional role and identity’, ‘Beliefs about capabilities’, ‘Memory, attention and decision processes’, ‘Environmental context and resources’ and ‘Social influences’ [9]. The CIFR has five main domains: ‘Intervention characteristics’, ‘Outer setting’, ‘Inner setting’, ‘Characteristics of individuals’ and ‘Process (of implementation)’. Constructs are grouped within these domains. For example, the domain ‘Outer setting’ contains the constructs ‘Patient needs and resources’, ‘Cosmopolitanism’, ‘Peer pressure’ and ‘External policy and initiatives’ [11]. Both frameworks include constructs of relevance to both individual and organisational level change [12]. However, the TDF has greater focus on determinants of behaviour change at the individual level, whilst the CFIR has greater focus on organisational-level constructs, and includes domains focussing on characteristics of interventions and implementation processes.

The HipHOP study contained embedded qualitative research which explored trial-related experiences and perceptions of patients, consultant orthopaedic surgeons and health professionals; those findings are reported elsewhere [3]. With surgeon participants, we also sought to understand potential barriers to implementation in practice of future trial findings; these findings are reported in the present paper. Consultant surgeons are surgical team leaders and are responsible for making clinical judgements about which surgical treatments to offer patients. It is therefore extremely important to consider their perspectives on implementation of evidence-based surgical guidance.

## Methods

### Design

Single, semi-structured qualitative interviews were conducted with consultant orthopaedic surgeons within HipHOP, a multi-centre, feasibility RCT [3]. Reporting follows the ‘Consolidated criteria for reporting qualitative research‘ (COREQ) guidance (Additional File 1) [13].

### Participants

Consultant orthopaedic surgeons were purposively sampled, ensuring inclusion of: surgeons based at the National Health Service (NHS) hospital sites who agreed for their patients to be included in the feasibility RCT; surgeons at the hospital sites who did not agree to their patients being included in the RCT; and surgeons from two additional NHS sites not involved in the feasibility study. We chose to recruit surgeons from these three groups because they were likely to have different levels of awareness of the HipHOP study and varying opinions on how implant types should be selected. Surgeons at hospital sites where the feasibility RCT was being conducted would have been introduced to the HipHOP study and would already have had to consider whether or not they would be willing for their patients to be randomised into the trial. Those who agreed to randomisation might be expected to be more open to considering alternative surgical approaches and to feel happier to follow external guidance on implant type than surgeons who had declined involvement in the trial. Surgeons from the additional NHS sites (sites which were not currently participating in the feasibility trial) had received less exposure to the HipHOP study and their perspectives on implementation might be expected to have more in common with the wider surgical population.

The intended sample size was 20–30 surgeons with a view to gaining a range of perspectives, within a dataset of manageable size for a meaningful, in-depth analysis. An exclusion criterion was having a hearing impairment which would impede telephone communication.

At each site, the Local Collaborator or Principal Investigator identified suitable surgeons. These surgeons were emailed the study information by TB (HipHOP Chief Investigator) before being contacted by the researcher (AD) and invited to participate.

### Data collection

One-to-one telephone interviews were conducted by AD. Participants were advised that only university-based research team members would have access to full interview transcripts so that clinician research team members (potential colleagues of participants) would not be able to identify participants.

Informed consent was audio-recorded immediately before the research interview; a separate audio-recording was created of the interview. The researcher made field notes following interviews.

Interviews were guided by an interview schedule which was developed for the HipHOP study. It was informed by relevant literature and reviewed by patient and public involvement contributors and clinical research team members. The interview schedule included topics related to trial feasibility, drawing on the Theoretical Framework of Acceptability [14]; further details and findings related to trial feasibility topics are reported elsewhere [3]. Participants’ thoughts regarding implementation of possible guidance from a definitive trial were explored (Table 1; for full interview schedule see Additional File 2).

**Table 1:** Interview schedule topics related to implementation issues

If the current feasibility study suggests we can run a full trial, the next stage of research will be a full trial. Depending on that trial’s findings, we may be recommending the use of one type of hip joint or the other.
1. How would you feel about being given guidelines recommending one approach or another?
2. How would you feel about following such guidelines?
3. What barriers do you think there might be to such recommendations being implemented in practice? a. How could these barriers be overcome
4. What would facilitate the implementation of any such recommendations into practice?

### Analysis

Interview audio-recordings were transcribed verbatim. An inductive, data-driven thematic analysis was conducted to explore and understand “patterns” within the data ([15], p79). An inductive rather than a deductive, theoretically-driven approach was taken because we sought to focus on the potential barriers to implementation of evidence-based guidance which were most salient to the surgeon participants.

We used the Framework approach to structure the analysis [16, 17]. Framework is a systematic approach with a transparency enabling other team members to follow and discuss the analysis and decisions made. Following Ritchie & Spencer (1994) [16], the five main analysis stages were: *Familiarisation* (transcripts were read and re-read, with notes made of apparently important issues); *Identifying a thematic framework* (ideas were organised into a ‘working thematic framework’ – a list of categories and sub-categories); *Indexing* (the working thematic framework was applied to transcripts, indicating location of categories and sub-categories within transcripts); *Charting* (matrices were created in which summaries of relevant data were presented by participant and category/sub-category), and *Mapping and interpretation* (charts were interrogated to further the interpretation of data and to gain deeper understanding, furthering the development of the final themes). See Additional File 3 for further details.

On completion of the inductive analysis, we related the findings to the TDF and CFIR. Mapping our findings onto theoretical constructs meant that it would be possible for our findings to be considered using labels which would be consistent with the wider literature, contributing to the wider evidence base. This mapping would also enable researchers to draw on theory and evidence when considering processes by which implementation of guidance might be optimised [9]. We considered both TDF and CFIR because we did not know, a priori, which issues might be most important in the present context and so could not predict which framework would be the best fit. Mapping our findings to theory after an inductive analysis would enable us to determine which framework would be the most useful going forward – or how the frameworks might be appropriately combined for the present context.

## Results

### Descriptive information

Forty consultant orthopaedic surgeons were invited to participate; 16 took part in an interview (Table 2). Reasons for non-responding were not recorded. Interviews took place May – August 2021.

**Table 2:** Participant and interview characteristics

Participant/interview characteristic	Value
Hospital site in relation to HipHOP feasibility trial*	
Trial recruitment site	*N* = 10
(‘Randomising’)	(*N* = 4)
(‘Non-randomising’)	(*N* = 6)
Interview-only site	*N* = 6
Number of hip replacement operations surgeons reported typically performing each month†	Median = 15.85 (range: ≤10 to ≥30) ‡
Number of years at consultant grade	Median = 14 (range: ≤5 to ≥25) ‡
Number of years practicing hip replacement surgery	Median = 16.5 (range: ≤10 to ≥30) ‡
Interview duration (minutes)	Median: 29 (range: 18 to 49)

*‘Randomising’: surgeons who consented to their patients being randomised within the feasibility trial; ‘non-randomising’: surgeons who did not consent to their patients being recruited to the feasibility trial; Interview-only site: surgeons were based at NHS hospital sites which did not recruit patients into the feasibility trial

†Where surgeons reported numbers as a range, mean average between the two figures was used

‡Exact minimum/maximum values are not presented to avoid identification of participants

### Analytic findings

Three themes were developed: ‘Beliefs about best practice’, ‘In my hands’ and ‘Ensuring competency’. To maintain participant confidentiality, participants are identified by letters, with indication of their grouping in the HipHOP feasibility study (randomising, non-randomising or interview-only site). To further enhance anonymity, indication of participants’ personal implant usage was removed from quotations. The working thematic framework relevant to this paper and the final thematic structure are detailed in Additional File 3.

#### Theme 1: beliefs about best practice

##### Perceptions informing current practice

Surgeons considered a range of topics when discussing implementing recommendations into practice. One factor appearing to influence willingness to change was the belief that changing practice would lead to better outcomes:

*I think what would make me change is someone demonstrating that there is a problem or there’s something that could be better than there currently is. And if that fitted with the procedures that I’m trained and comfortable to do and that fitted with my local group of patients and their outcomes following the change or their PROMs [Patient Reported Outcome Measures] scores let’s say then that’s fine.* (Surgeon B, interview-only site)

Within interviews, there was discussion around surgeons’ current approaches to implant types. It is useful to reflect on these discussions as they illustrate perceptions potentially contributing to beliefs about best practice and clinical decisions. Reported practice varied, from currently using only or mostly cemented implants, through using more even numbers of cemented and hybrid implants, to using mostly hybrid implants. Many participants seemed to weigh up a range of factors for each individual patient when deciding which implant to use. Factors related to bone density (particularly older age and being female) and anatomical issues seemed important to many. Surgeons typically preferred to use cemented implants in individuals likely to have lower bone density (particularly older patients), citing concerns about fracture risk, and hybrid implants in those with higher bone density (e.g. younger patients). However, age thresholds reported to influence decisions varied. It seemed that surgeons might feel most comfortable about changing practice for patients in the middle age range:

*I think the younger the patients*,* the more the hard sell it would be as a trial*,* or the results of that trial*,* if they went against the hybrid.* (Surgeon T, non-randomising)

Beliefs about the current evidence base with respect to use of different implant types and various outcomes were shared when discussing implant types:

*So the [implant type A]*,* […] that’s my preferred option. And that’s really born out of National Joint Register and certain studies*,* which have shown that the overall functional outcomes and longevity of the construct that I use seems to be the best performing.* (Surgeon C, randomising)

*the vast majority of my hips have been [implant type B]. And the reason being the data*,* the evidence*,* and the versatility of the hip.* (Surgeon R, interview-only site)

The National Joint Registry (NJR) is an implantable medical device registry covering England, Wales, Northern Ireland, the Isle of Man and Guernsey. It collects information to monitor joint replacement implants’ performance. It seems to be an important resource for surgeons using implants, and was seen to support practice focusing on either implant type.

It appeared that surgeon participants considered a range of factors, including available evidence, when considering implant choice. However, there seemed to be variability in resulting opinions around current best practice.

##### Evidence base informing future practice

Having good quality evidence with clear indication of best practice seemed important to participants when future practice recommendations were considered. Some suggested that they would consider changing their practice if presented with clear trial evidence:

*I hope it [the trial] would give me and my colleagues*,* future colleagues*,* irrefutable evidence as to what the benefits are. And then I would change my practice accordingly.* (Surgeon A, non-randomising)

*Well*,* if the trial – clearly if the trial showed strong evidence one way or the other*,* then you’ve got to act on that evidence. And*,* you know*,* it’s better that guidelines is based on good evidence than on dogma or*,* you know*,* poor levels of evidence*. (Surgeon T, non-randomising)

Some participants were cautious when considering the impact of trial evidence on surgical practice. There was wariness of poorly conducted trials, and a need to be convinced that a trial was of good quality before considering changing practice.

*Well*,* if the evidence was clear-cut and I believe the findings of the study and the way it was reported and the data reported*,* then I wouldn’t have any problems at all [in following guidelines]. If I had – if I disagreed with the way the study was reported*,* or over-generalisation of its findings*,* then I might have a problem* (Surgeon O, randomising).

Some seemed to be sceptical as to how conclusive a trial’s findings could be. There was a belief that a very long follow-up period would be needed to identify differences between the implants, and a concern as to whether a study could be adequately powered to detect a difference. It appeared that evidence from a good quality trial which showed a clear difference between implant types would be most likely to convince surgeons to consider changing practice:

*I suppose it depends how strongly the results*,* you know*,* come out [inaudible] one or the other. So my worry is that you won’t really see that difference for many*,* many years.* (Surgeon U, randomising)

*the problem is you won’t come out with an absolute conclusion. You won’t get the numbers to do that. You’ll get a signal. And so*,* it will be advisory*. (Surgeon J, interview-only site)

*I’d be surprised if it [a trial] could find an obvious difference and I’d be equally surprised if that influenced a certain number of surgeons. Because*,* you know*,* I think most of us rightly so carry out operations that we’re trained to do. And most of us would not change our practice unless we’d identified a definite problem or something that could be definitely improved upon.* (Surgeon B, interview-only site)

Nevertheless, some surgeons considered a trial which detected only a small difference to be valuable because of perceived differences in costs between the two implants:

*It’s more expensive to do a hybrid*,* […] it means we can use a cemented implant with a very good track record that’s much less expensive. And if you kind of extrapolate that to the NHS there’s a massive*,* massive difference in cost to the NHS and particularly in kind of current economic climate.* (Surgeon R, interview-only site)

There was not full consensus that cemented implants are cheaper overall, but it seems surgeons may consider information about cost outcomes when implementing guidance.

Some participants seemed to perceive that providing evidence might not be sufficient to change some surgeon’s opinions regarding best practice. There was a sense that some individuals would behave in line with personal beliefs about optimal treatments which might not be evidence-based:

*we can be quite self-opinionated. […] the challenge will be preconceived biases of what they believe works best*,* not always based on sound science.* (Surgeon A, non-randomising)

*people will always interpret the results of research from their own point of view. And there is*,* you know*,* all sorts of compelling evidence out there which is ignored quite regularly […] By me as much as anybody else*. (Surgeon T, non-randomising)

##### Dissemination of trial findings and resulting recommendations

When discussing how to facilitate implementation, many participants considered how trial findings might most effectively be shared with surgeons. The need to engage with surgeons in a way suited to them was raised:

*I mean it has to be data presented in the appropriate channels*,* meetings*,* publications etc.*,* […] But it just has to be presented in the right way at the right place to people.* (Surgeon G, non-randomising)

Dissemination by familiar and respected organisations such as the British Hip Society and the British Orthopaedic Association (BOA) seemed to be potentially influential. Attention from NICE (The National Institute for Health and Care Excellence) was also deemed valuable:

*the evidence and the support of national governing bodies would probably be helpful.* (Surgeon Z, randomising)

*if there was [inaudible] guidelines and it was recommended by – for example*,* if NICE recommended it then*,* yeah*,* [inaudible] take notice of it I think*. (Surgeon C, randomising)

*So if the message is there to the people who*,* hip surgeons*,* most of whom are part of the British Hip Society or hip surgeons generally are mostly part of the BOA*,* if you can take that message through something like that*,* then you’d hope that most people would say well there is the evidence*,* and I should think about changing what I’ve done. […] So journals are good but not everybody reads a journal*,* they sometimes don’t.* (Surgeon M, interview-only site)

It might be important to seek change at the group/team level rather than focussing on individual change in practice. One participant mentioned an awareness of risk if a surgeon’s practice differed from their peers:

*So I do end up using [implant type] in the [age group] patients*,* partly via peer pressure I guess […] it’s common practice […]*,* and you have to slightly bow to that*,* if you see what I mean? […] it’s better to keep your practice core if you don’t – you know*,* to stand out with practice that’s not so common*,* it’s not necessarily a good idea.* (Surgeon V, interview-only site)

Whilst participants seemed keen that practice recommendations be based on good quality evidence, a desire to still have autonomy to use clinical judgement for individual cases was raised in some interviews.

*We’re used to guidelines. And we generally follow them. But we are very keen they are called guidelines*,* not rules because there are always exceptions.* (Surgeon J, interview-only site)

It was not specified what types of ‘exceptions’ this participant had in mind as leading to reluctance to follow guidelines. However, an issue which received discussion was that it is possible that while one approach might, in general, lead to optimum outcomes and become recommended in evidence-based guidelines, an important factor determining outcomes for an individual patient is the skill and experience of the specific surgeon. This issue is explored within Theme 2.

#### Theme 2: in my hands

When discussing their current practice, in addition to considering the wider evidence base, participants also considered their own personal results:

*Well*,* the thing is my results with [implant type 1] are so good. I don’t think I’ll be able to improve on that using [implant type 2].* (Surgeon N, non-randomising)

*I’m looking at my practice and seeing a very*,* very low complication rate. [Implant type is] very user friendly*,* it’s very predictable*,* quick and it’s very teachable to trainees […] You know when something works in life*,* […] you start to think why would I want to change something that I see work?* (Surgeon D, non-randomising)

An issue raised by participants was that orthopaedic surgery can be considered a ‘craft’ speciality. A successful hip replacement could be dependent on an individual surgeon’s skill level with a specific procedure. Participants raised the possibility that research evidence could conflict with an individual surgeon’s results because of personal training and experience:

*their results might not be – might be different to what they think that the study shows*,* whether it’s directly comparable or not. So I think it’s a – it might*,* in isolation*,* be quite difficult to get people to make the change based on – just on the basis of this study.* (Surgeon U, randomising)

*one of the frequently stated barriers to surgeon’s changing practice […] is that they don’t feel adequately trained […] and they’re concerned that whilst the registry data might suggest that’s better that they would have worse – personally have worse outcomes because that’s not – they would be using – adopting a new technique basically.* (Surgeon O, randomising)

Participants appeared to be reluctant to consider a change which could damage good personal records:

*I don’t want to change now because of my results really. I’ve got results to prove on the National Joint Registry*,* so I think I won’t be very keen to change* [and later:] *At the end of the day it’s what works in your hands. And I think surgeons would be probably sticking to that really*,* rather than following guidelines.* (Surgeon N, non-randomising)

*your results are based upon what you’re good at. And then someone’s telling you to change something that you’re good at to something you’re not good at then you’re getting crappy results. […] if the main trial proves one over the other*,* I think you’ll still find it hard to convince surgeons who do a totally different type of replacement and have had very*,* very good results on the registry and have them for a number of years to change.* (Surgeon C, randomising)

There may be two related issues influencing willingness to implement recommendations. First, concerns related to individual surgeons’ insufficient training or experience leading to poor results were clearly apparent. Second, surgeons may hold beliefs around which approach they personally are best at or find easier, being more confident with one approach, or feeling reluctant to change from familiar practice:

*So many surgeons only really do uncemented sockets and claim that it’s too difficult to do a cemented socket* (Surgeon V, interview-only site).

*you have many surgeons […] who have only done cemented and would just not be willing to change.* (Surgeon G, non-randomising)

Nevertheless, a desire to follow evidence could be seen, even when accompanied by concerns around changing practice:

*Switching to an implant that I’m not normally using*,* and a different technique if you like*,* would bring a little anxiety with it. But no*,* if there was clear evidence then that that was it*,* then I would.* (Surgeon G, non-randomising)

Some surgeons seemed comfortable in using both implant types, and for such surgeons a change in practice might be straightforward:

*I already do both types of hips*,* so if someone said well actually if you did more these this is*,* will be a better outcome*,* or this will be a better whatever*,* and I’d have to say well I could do both*,* let’s do the one where the results are better.* (Surgeon M, interview-only site)

#### Theme 3: ensuring competency

Participants’ training background seemed influential in their preferences and confidence with using implant types:

*So my actual final decision on why to only use [implant type] was therefore based on my final year of fellowship at [hospital name] when I did all [implant type]. And I just personally felt comfortable doing it*,* the data supported it*,* I felt*,* on the NJR*,* so I stuck with it.* (Surgeon G, non-randomising) 

*Yes*,* so there are a number of reasons [for currently preferring to do implant type]. I guess partly influenced by my training.* (Surgeon O, randomising)

For surgeons to be willing to change practice, the need for training was raised:

*if you’re trying to get surgeons to switch from one established practice to the one they don’t have an established practice in then that can be a training issue.* (Surgeon O, randomising)

*if you’re asking people to do things that they aren’t trained to do or haven’t done for many years then probably that’s may not be for the greater good.* (Surgeon B, interview-only site)

Surgeon B’s quote highlights that ensuring surgeons are suitably trained and experienced before asking them to change practice is important to ensure that patients do not suffer in a transition process.

However, challenges around implementing training were identified. Some participants thought that some surgeons might not wish to carry out training designed to enable practice change:

*some surgeons may say*,* “I don’t use this implant*,* I don’t know how to use it. I’m not familiar with it*,*” and may not want to retrain or use it.* (Surgeon Q, non-randomising)

One surgeon perceived the best approach would be for training to target new surgical trainees, teaching them the approach favoured by evidence, rather than to retrain existing, qualified surgeons:

*The only way that barriers can be changed is if the newer bunch of surgeons*,* you know*,* the newer registrars*,* trainees*,* coming through rank at an early age were trained only in that type of implant. […] that would have to come from almost the Department of Health*,* I guess.* [and later:] *So*,* bringing it into the – bringing it into the initial training*,* the curriculum. But that would have to be driven from a much higher level.* (Surgeon C, randomising)

This quote highlights the complexity of introducing changes in surgical training provision. As surgical training currently takes an apprenticeship approach, with ‘new’ surgeons receiving training from ‘experienced’ surgeons, ‘new’ surgeons’ learning is limited by the ‘experienced’ surgeons’ expertise. Training new surgical trainees in a different surgical approach would therefore require a change in training structure and delivery.

One surgeon proposed an alternative strategy to training: if different groups of patients did better with different implant types, then surgical provision could be organised so that surgeons comfortable with specific procedures are allocated patients who are best suited to those procedures:

*if you identify a [patient] group that are definitely better off having a hybrid than a cemented then maybe they could be done by surgeons who are comfortable doing that or*,* you know*,* who are willing to do those procedures.* (Surgeon B, interview-only site)

### Relating findings to TDF and CFIR

See Additional File 4 for detailed consideration of how themes identified inductively were related to the domains or constructs of the TDF and CFIR, with figures mapping themes to constructs, when the two frameworks were considered separately. Both frameworks appeared to be of relevance to the present implementation context, with their respective focuses allowing elaboration of different issues identified within the inductive analysis. Here we consider the most salient ways in which factors in these frameworks appeared to fit with issues identified through inductive analysis.

The TDF was valuable because it considers a range of determinants to individual behaviour change explicitly, and a number of those determinants appeared to be of particular importance here. The TDF domains ‘Knowledge’ and ‘Belief about consequences’ mapped onto the need for surgeons to know what the evidence and guidelines are, and their beliefs about what would happen if they followed those guidelines. For example, whether surgeons believe that following guidance would mean better patient outcomes, or believe that patient outcomes would worsen because of a learning curve in adopting a new approach. The ‘Skills’ domain seemed highly relevant to our findings about surgeon competency to use a recommended implant, and ‘Beliefs about capabilities’ related to surgeons’ confidence in using an implant.

The CFIR usefully makes explicit features of the specific intervention. From the domain 'Intervention characteristics', ‘Evidence strength and quality’ seemed important to participants, and the construct ‘Relative advantage’ also appeared relevant: surgeons could be considering the benefits and risks of using one implant, recommended in evidence-based guidelines, against another implant which they might have chosen using clinical judgement. These two constructs would seem to be useful in considering factors underlying the TDF’s ‘Beliefs about consequences’.

Both frameworks allow for consideration of social pressures, with influence and behaviours of colleagues and respected professional organisations fitting within ‘Peer pressure’ and ‘Social influence’ within the CFIR and TDF respectively.

The need for training to be provided to support change in practice would fit within ‘Environmental context and resources’ (TDF) and ‘External policies and incentives’ (CFIR). These factors would also seem relevant to the need for recommendation development and dissemination.

Overall, the TDF, with its specification of determinants of individual behaviour change appeared to be particularly useful to support implementation of guidance around implant usage. However, the CFIR had value in aiding detailed evaluation within some areas.

## Discussion

This qualitative study highlighted the importance of guidelines being based on good quality evidence, showing a clear difference between implant types, and endorsed by respected professional organisations to support implementation of implant recommendations by surgeons. It appeared that surgeons would also need to believe that changing surgical approach would have a positive impact on their personal results, recognising individual differences in training, experiences and skills. The importance of enabling surgeons to gain required skills was identified.

Previous research has identified that multiple levels of evidence can be influential when considering guideline implementation, with ‘micro’ level evidence (including training and learning from personal experience), alongside ‘meso’ and ‘macro’ evidence levels (including influence of colleague surgeons and research evidence) as having potential to influence decision-making [18, 19]. The ‘Getting it Right First Time’ (GIRFT) review of orthopaedic surgery identified that implant selection can be influenced by surgeons’ training location and accepted local practices [20].

Using professional bodies was identified as a strategy for disseminating guidance. Previous research has identified that advice from societies of fellow professionals such as the BOA seem more likely to be accepted and trusted than sources with less surgeon input [21]. The GIRFT report advocates for professional organisations developing evidence-based guidance to support decision making on implant fixation [20].

Concerns about potential negative impact of adopting a new procedure were identified in the present study. Improvements in operation duration and surgical outcomes have been evidenced as surgeons’ experience with a procedure increases, during a ‘learning curve’ period [22, 23]. Our findings identified the importance of ensuring surgeons are appropriately trained and experienced if they are expected to follow evidence-based guidance recommending change in practice, although how this may best be achieved is not yet clear. Further research including a wider range of perspectives on this issue, including professional bodies such as Health Education England and the Joint Committee on Surgical Training, is likely to provide useful insight. In evaluating implementation of GIRFT recommendations around fixation, barriers to practice change identified were financial costs and organisational issues related to meeting staff training needs [24].

Both TDF and CFIR theory-based frameworks were found to be of relevance to the current implementation context, with each allowing elaboration of particular issues. The TDF domains ‘Knowledge’, ‘Beliefs about consequences’, ‘Skills’ and ‘Beliefs about capabilities’ seemed salient in our findings, and the CFIR constructs ‘Evidence strength and quality’ and ‘Relative advantage’ appeared relevant to support the consideration of factors underlying ‘Beliefs about consequences’. Both frameworks enable consideration of social pressures, training and dissemination needs. With its focus on determinants of individual behaviour change, the TDF seemed to be particularly useful when considering implementation of implant fixation guidance. However, the present study focussed on one specific stakeholder group, whose individual behaviour would be essential to implementation. Further research is needed to explore the perspectives of other stakeholders – such as professional organisations, health services managers, surgical training leads – and to consider more deeply how implementation should be conducted. In exploring such perspectives, the CFIR’s explicit elaboration of the domains ‘Outer setting’, ‘Inner setting’ and ‘Process’ may increase in relevance.

### Implications

The present findings suggest that, for surgeons to implement fixation-related hip implant guidance, surgeons need to believe that they are using the approach that will lead to optimum outcomes. To persuade surgeons that guidance is trustworthy, good quality evidence and endorsement by professional organisations appear important. To ensure that surgeons are confident in using the recommended approach, opportunities to train and gain experience to ensure that they, as individuals, can gain good results using the evidence-based approach may be needed.

### Strengths and limitations

In interviewing 16 surgeons, our sample was smaller than the intended sample size of 20–30 participants. However, within this sample we had good representation of surgeons across HipHOP study recruitment sites and background, and the sample size proved sufficient to achieve our aims of obtaining a range of perspectives and developing a meaningful analysis.

We only considered surgeons's perspectives in the present analysis. Other perspectives are likely to be important in optimising implementation of implant fixation guidance, e.g. hospital management, guideline developers, and surgical training leads. For example, hospitals having other, urgent priorities was identified as a barrier to implementing GIRFT recommendations [24]. Gaining perspectives of such groups may identify additional relevant theoretical factors to consider when developing implementation interventions.

Our interviews discussed guidelines at a hypothetical stage. Until a full trial is completed, it is unclear what recommendations might result. Theoretical constructs which more closely focus on processes around implementation might therefore not have been salient at the time of interviews, but may be viewed as of more importance later. Nevertheless, it is valuable to have gained surgeons’ perspectives, and identify some issues that are likely to be important so that they can be considered and addressed in parallel with RCTs evaluating effectiveness.

A theory-based framework was not used in designing our interview schedule nor structuring analysis. This approach may allow topics to be raised which might not neatly fit within a theory-based framework [25]. Within the time constraints of an interview with additional purposes, we used broad questions with the aim of identifying topics surgeons perceived as being important and conducted an inductive analysis with the aim of drawing out the most salient issues from surgeons’ perspectives. This was valuable in allowing a thoughtful and nuanced understanding to be developed, grounded in participants’ views and experiences. Nevertheless, prior to implementation of implant recommendations, further exploration specifically targeting constructs identified by a theory-based framework, using approaches such as those described by Atkins et al. [26], across stakeholder groups, might highlight additional relevant issues.

## Conclusions

The implementation of recommendations for hip implant fixation type is complex because of the need for surgeons to have an appropriate level of skill to avoid suboptimal surgical outcomes. Basing recommendations on good quality evidence and gaining endorsement of recommendations by respected professional organisations seems likely to encourage practice change. However, training and experience in the recommended approach should be made available to surgeons, such that they are confident that a change in practice will lead to improvements in personal surgical results.

## Supplementary Information

Additional File 1. COREQ checklist from Table 1 in Tong A, Sainsbury P, & Craig J (2007). Completed COREQ checklist for this paper.

Additional File 2. Surgeon interview schedule. Full interview schedule used for interviews with consultant orthopaedic surgeons.

Additional File 3. Further analysis information. 1) Additional details on analysis process; 2) Working thematic framework (implementation categories); 3) Final thematic structure of implementation findings.

Additional File 4. Mapping findings to theory-based frameworks. Document relating study findings to the theoretical domains identified within the Theoretical Domains Framework (Cane et al, 2012; Michie et al, 2005) and the constructs identified within the Consolidated Framework for Implementation Research (Damschroder et al, 2009).

## Data Availability

Restrictions apply to the availability of the dataset in order to protect participants’ identity, in line with the study’s ethical approval. Contact the corresponding author for information.

## Abbreviations

BOA: British Orthopaedic Association
CFIR: Consolidated Framework for Implementation Research
GIRFT: Getting it Right First Time
HipHOP: HIP arthroplasty with Hybrid Or cemented implants: Patient reported outcomes
NHS: National Health Service
NICE: The National Institute for Health and Care Excellence
NJR: National Joint Registry
PROMs: Patient Reported Outcome Measures
RCT: Randomised controlled trial
TDF: Theoretical Domains Framework
THR: Total hip replacement
