# Supplementary material for: Implementation of hip replacement surgery recommendations: a qualitative study of orthopaedic surgeons’ perspectives
Source: BMC Musculoskelet Disord. 2025 Dec 8;27:26. doi: 10.1186/s12891-025-09334-z (PMC12797885; doi:10.1186/s12891-025-09334-z)
Supplement: Supplementary file 1 — Additional File 1. COREQ checklist from Table 1 in Tong A, Sainsbury P, & Craig J (2007). Completed COREQ checklist for this paper. [file 12891_2025_9334_MOESM1_ESM.docx]

**Additional File 1: COREQ checklist from Table 1 in Tong, A., Sainsbury, P., & Craig, J. (2007).**

| **Number** | **Item** | **Guide question/description** | **Location in paper** |
| --- | --- | --- | --- |
| **Domain 1: Research team and reflexivity**  *Personal characteristics* | | | |
| 1 | Interviewer/facilitator | Which author/s conducted the interview or focus group? | Data collection |
| 2 | Credentials | What were the researcher’s credentials? E.g. PhD, MD | Additional File (AF) 3 |
| 3 | Occupation | What was their occupation at the time of the study? | AF3 |
| 4 | Gender | Was the researcher male or female? | AF3 |
| 5 | Experience and training | What experience or training did the researcher have? | AF3 |
| *Relationship with participants* | | | |
| 6 | Relationship established | Was a relationship established prior to study commencement? | AF3 |
| 7 | Participant knowledge of the interviewer | What did the participants know about the researcher? E.g. personal goals, reasons for doing the research? | Data collection |
| 8 | Interviewer characteristics | What characteristics were reported about the interviewer? E.g. bias, assumptions, reasons and interests in the research topic | Data collection |
| **Domain 2: Study design**  *Theoretical framework* | | | |
| 9 | Methodological orientation and theory | What methodological orientation was stated to underpin the study? E.g. grounded theory, discourse analysis, ethnography, phenomenology, content analysis | Analysis |
| 10 | Sampling | How were the participants selected? E.g. purposive, convenience, consecutive, snowball | Participants |
| 11 | Method of approach | How were participants approached? E.g. face-to-face, telephone, mail, email | Participants |
| 12 | Sample size | How many participants were in the study? | Results |
| 13 | Non-participation | How many people refused to participate or dropped out? Reasons? | Results |
| *Setting* | | | |
| 14 | Setting of data collection | Where was the data collected? E.g. home, clinic, workplace | Data collection |
| 15 | Presence of non-participants | Was anyone else present besides the participants and researchers? | Data collection |
| 16 | Description of sample | What were the important characteristics of the sample? E.g. demographic data, date | Results |
| *Data collection* | | | |
| 17 | Interview guide | Were questions, prompts, guides provided by the authors? Was it pilot tested? | Data collection & AF 2 |
| 18 | Repeat interviews | Were repeat interviews carried out? If yes, how many? | Design |
| 19 | Audio/visual recording | Did the research use audio or visual recording to collect the data? | Data collection |
| 20 | Field notes | Were field notes made during and/or after the interview or focus group? | Data collection |
| 21 | Duration | What was the duration of the interviews or focus groups? | Results |
| 22 | Data saturation | Was data saturation discussed? | Saturation was not a pre-specified goal within this sample and its validity as a goal for sampling is questioned within the field. Rationale for intended sample size is provided (Participants) |
| 23 | Transcripts returned | Were transcripts returned to participants for comment and/or correction? | Analysis |
| **Domain 3: Analysis and findings**  *Data analysis* | | | |
| 24 | Number of data coders | How many data coders coded the data? | Analysis |
| 25 | Description of the coding tree | Did authors provide a description of the coding tree? | AF3 |
| 26 | Derivation of themes | Were themes identified in advance or retrieved from the data? | Analysis & AF3 |
| 27 | Software | What software, if applicable, was used to manage the data? | AF3 |
| 28 | Participant checking | Did participants provide feedback on the findings? | AF3 |
| *Reporting* | | | |
| 29 | Quotations presented | Were participant quotations presented to illustrate the themes/findings? Was each quotation identified? E.g. participant number | Results |
| 30 | Data and findings consistent | Was there consistency between the data presented and the findings? | Results |
| 31 | Clarity of major themes | Were major themes clearly presented in the findings? | Results |
| 32 | Clarity of minor themes | Is there a description of diverse cases or discussion of minor themes? | Results |

Note: AF = Additional File.

**Reference:**

Tong, A., Sainsbury, P., & Craig, J. (2007). Consolidated criteria for reporting qualitative research (COREQ): a 32-item checklist for interviews and focus groups. *International Journal for Quality in Health Care, 19*, 349-357. https://doi.org/10.1093/mzm042
