# Supplementary material for: Implementation of hip replacement surgery recommendations: a qualitative study of orthopaedic surgeons’ perspectives
Source: BMC Musculoskelet Disord. 2025 Dec 8;27:26. doi: 10.1186/s12891-025-09334-z (PMC12797885; doi:10.1186/s12891-025-09334-z)
Supplement: Supplementary file 2 — Additional File 2. Surgeon interview schedule. Full interview schedule used for interviews with consultant orthopaedic surgeons. [file 12891_2025_9334_MOESM2_ESM.docx]

**Additional File 2: Surgeon interview schedule**

**Note:** The interview schedule could be adapted as interviews progressed – should participants raise issues of importance that the research team had not anticipated prior to commencing interviews, those topics would be added to the interview schedule so that later participants would be asked about those issues.

**RECRUITING SITE SURGEONS; PARTICIPATING IN HIPHOP (ALLOWING PATIENTS TO BE RECRUITED AND RANDOMISED)**

**Thoughts about the research topic**

1. Could you tell me what you understand the HipHOP trial to be about?
2. What do you think about fully cemented hip arthroplasty?
   1. Could you tell me about your experience of working with these implants during surgery?
   2. Could you tell me about your experience of complications when working with these implants?
   3. How do you find patients typically do after this type of surgery? *(prompts: pain, recovery of usual activities, range of movement)*
   4. Any other benefits or risks to using fully cemented implants?
3. What do you think about hybrid hip arthroplasty?
   1. Could you tell me about your experience of working with these implants during surgery?
   2. Could you tell me about your experience of complications when working with these implants?
   3. How do you find patients typically do after this type of surgery? *(prompts: pain, recovery of usual activities, range of movement)*
   4. Any other benefits or risks to using hybrid hip implants?
4. How would you usually decide whether to use one approach or another with an individual patient?
5. Which of these approaches would typically be your preferred option? – why is that? *(prompts: personal experience outcomes? Research evidence? Influence of other surgeons? Training? Institutional issues/expectations? Cost?)*
6. To what extent do you feel there is equipoise – i.e. to what extent do you feel that we do not know which of these treatments is best?
   1. Does running a trial to compare these two implants make sense to you?
   2. Which type of treatment do you think a trial would find to be the most effective?
7. How ethical do you think it is to allocate patients to one type of implant or the other using randomisation?
8. What information would you usually give to patients about the types of implant available? *(Prompts: would you describe the type of implant? Would you indicate that more than one type is available? Do patients ask about the type of implant?)*

**Surgeon’s role in the research**

1. Could you tell me about any experience of research you’ve had previously? *(Prompts: as someone leading a research project, collaborating, or talking to patients to a research project)*
2. How important do you think it is for clinicians to take part in research?
3. Could you tell me about the role you currently have with the HipHOP feasibility trial?
   1. Have you had any conversations with patients about the trial? If yes:
   2. How do you think patients have felt about the study? *(prompts: have they expressed any concerns? Could you tell me about those?)*
   3. How have you found these conversations? *(prompts: any difficulties? How confident felt about talking about the study?)*
4. What influenced your decision to allow your patients to be randomised for this study?
5. How do you feel about your patients being randomised to receive one treatment or the other?
6. How do you think patients feel about being randomised to one treatment or another? Are you aware of their having any concerns about this aspect of taking part? What concerns do you think they might have?
7. How do you feel about carrying out a type of surgery according to a study protocol rather than according to your own clinical decision?
8. Have you experienced any problems in taking part in the study so far?
   1. How could these problems be overcome?

If the feasibility study is successful, the next step would be a full trial to determine which treatment is the most effective.

1. Would you also be happy to take part in a full trial – by allowing your patients to be randomised and by providing the treatment indicated by the study protocol? Why is that?
2. How do you think your patients would feel about being randomised to one treatment or another in a full trial?
3. What concerns might you have about your patients being randomised in a full trial?
4. Thinking about the patients that you see, are there any patient characteristics that would make you unwilling for them to be allocated to one type of implant or another by randomisation?
5. How would carrying out treatment according to study protocol affect your day-to-day working?

In the full trial, we anticipate that the surgeon would be the person who first discusses the trial with a patient before sending the patient to a research nurse to fully consent them to take part in the study (if appropriate).

1. How would you feel about discussing the trial with a patient?
   1. Would you anticipate any problems with this? *(Prompts e.g. time, patient relationship, costs?)*
   2. How confident would you feel about discussing the trial with a patient?
   3. What training do you feel you would need to enable you to have such a discussion?
2. How do you think patients might feel about being randomised to one treatment or another within a full trial?
3. What concerns do you think they might have? How might such concerns best be addressed?
4. Are there any other problems that you would foresee in recruiting patients at your hospital to take part in the trial?

**Implementation issues**

If the current feasibility study suggests we can run a full trial, the next stage of research will be a full trial. Depending on that trial’s findings, we may be recommending the use of one type of hip joint or the other.

1. How would you feel about being given guidelines recommending one approach or another?
2. How would you feel about following such guidelines?
3. What barriers do you think there might be to such recommendations being implemented in practice?
   1. How could these barriers be overcome?
4. What would facilitate the implementation of any such recommendations into practice?

**Demographic questions**

- Gender
- Year first appointed as a consultant surgeon (or no. years since appointed as a consultant)
- For how many years have you been carrying out hip replacements?
- How many such operations would you typically carry out each month?

**RECRUITING SITE SURGEONS; NOT PARTICIPATING IN HIPHOP (HAVE DECLINED)**

**Thoughts about the research topic**

1. Could you tell me what you understand the HipHOP trial to be about?
2. What do you think about fully cemented hip arthroplasty?
   1. Could you tell me about your experience of working with these implants during surgery?
   2. Could you tell me about your experience of complications when working with these implants?
   3. How do you find patients typically do after this type of surgery? *(prompts: pain, recovery of usual activities, range of movement)*
   4. Any other benefits or risks to using fully cemented implants?
3. What do you think about hybrid hip arthroplasty?
   1. Could you tell me about your experience of working with these implants during surgery?
   2. Could you tell me about your experience of complications when working with these implants?
   3. How do you find patients typically do after this type of surgery? *(prompts: pain, recovery of usual activities, range of movement)*
   4. Any other benefits or risks to using hybrid hip implants?
4. How would you usually decide whether to use one approach or another with an individual patient?
5. Which of these approaches would typically be your preferred option? – why is that? *(prompts: personal experience outcomes? Research evidence? Influence of other surgeons? Training? Institutional issues/expectations? Cost?)*
6. To what extent do you feel there is equipoise – i.e. to what extent do you feel that we do not know which of these treatments is best?
   1. Does running a trial to compare these two implants make sense to you?
   2. Which type of treatment do you think a trial would find to be the most effective?
7. How ethical do you think it is to allocate patients to one type of implant or the other using randomisation?
8. What information would you usually give to patients about the types of implant available? *(Prompts: would you describe the type of implant? Would you indicate that more than one type is available? Do patients ask about the type of implant?)*

**Surgeon’s role in the research**

1. Could you tell me about any experience of research you’ve had previously? *(Prompts: as someone leading a research project, collaborating, or talking to patients to a research project)*
2. How important do you think it is for clinicians to take part in research?
3. I understand that some surgeons at your hospital are taking part in the HipHOP feasibility study, but you’ve chosen not to be involved – is that right?
4. Could you tell me the reasons for this? *(Prompt: what were your concerns about being involved with the study? What do you feel are the barriers to taking part in the feasibility study?)*
5. If the feasibility trial is successful, the next stage would be a full trial. What would help to overcome any barriers to taking part for the full trial?

If the feasibility study is successful, the next step would be a full trial to determine which treatment is the most effective.

1. How would you feel about your patients being randomised to receive one treatment or the other?
   1. How do you think patients would feel about being randomised to one treatment or another?
2. How would you feel about carrying out a type of surgery according to a study protocol rather than according to your own clinical decision?
3. What concerns might you have about your patients being randomised in a full trial?
4. Thinking about the patients that you see, are there any patient characteristics that would make you unwilling for them to be allocated to one type of implant or another by randomisation?
5. How would carrying out treatment according to study protocol affect your day-to-day working?

In the full trial, we anticipate that the surgeon would be the person who first discusses the trial with a patient before sending the patient to a research nurse to fully consent them to take part in the study (if appropriate).

1. How would you feel about discussing the trial with a patient?
   1. Would you anticipate any problems with this? *(Prompts e.g. time, patient relationship, costs?)*
   2. How confident would you feel about discussing the trial with a patient?
   3. What training do you feel you would need to enable you to have such a discussion?
2. How do you think patients might feel about being randomised to one treatment or another within a full trial?
3. What concerns do you think they might have? How might such concerns best be addressed?
4. Are there any other problems that you would foresee in recruiting patients at your hospital to take part in the trial?

**Implementation issues**

If the current feasibility study suggests we can run a full trial, the next stage of research will be a full trial. Depending on that trial’s findings, we may be recommending the use of one type of hip joint or the other.

1. How would you feel about being given guidelines recommending one approach or another?
2. How would you feel about following such guidelines?
3. What barriers do you think there might be to such recommendations being implemented in practice?
   1. How could these barriers be overcome?
4. What would facilitate the implementation of any such recommendations into practice?

**Demographic questions**

- Gender
- Year first appointed as a consultant surgeon (or no. years since appointed as a consultant)
- For how many years have you been carrying out hip replacements?
- How many such operations would you typically carry out each month?

**SURGEONS FROM NON-RECRUITING SITES**

**Thoughts about the research topic**

1. Could you tell me what you understand the HipHOP trial to be about?
2. What do you think about fully cemented hip arthroplasty?
   1. Could you tell me about your experience of working with these implants during surgery?
   2. Could you tell me about your experience of complications when working with these implants?
   3. How do you find patients typically do after this type of surgery? *(prompts: pain, recovery of usual activities, range of movement)*
   4. Any other benefits or risks to using fully cemented implants?
3. What do you think about hybrid hip arthroplasty?
   1. Could you tell me about your experience of working with these implants during surgery?
   2. Could you tell me about your experience of complications when working with these implants?
   3. How do you find patients typically do after this type of surgery? *(prompts: pain, recovery of usual activities, range of movement)*
   4. Any other benefits or risks to using hybrid hip implants?
4. How would you usually decide whether to use one approach or another with an individual patient?
5. Which of these approaches would typically be your preferred option? – why is that? *(prompts: personal experience outcomes? Research evidence? Influence of other surgeons? Training? Institutional issues/expectations? Cost?)*
6. To what extent do you feel there is equipoise – i.e. to what extent do you feel that we do not know which of these treatments is best?
   1. Does running a trial to compare these two implants make sense to you?
   2. Which type of treatment do you think a trial would find to be the most effective?
7. How ethical do you think it is to allocate patients to one type of implant or the other using randomisation?
8. What information would you usually give to patients about the types of implant available? *(Prompts: would you describe the type of implant? Would you indicate that more than one type is available? Do patients ask about the type of implant?)*

**Surgeon’s role in the research**

1. Could you tell me about any experience of research you’ve had previously? *(Prompts: as someone leading a research project, collaborating, or talking to patients to a research project)*
2. How important do you think it is for clinicians to take part in research?

If the feasibility study is successful, the next step would be a full trial to determine which treatment is the most effective.

1. How would you feel about your patients being randomised to receive one treatment or the other?
   1. How do you think patients would feel about being randomised to one treatment or another?
2. How would you feel about carrying out a type of surgery according to a study protocol rather than according to your own clinical decision?
3. What concerns might you have about your patients being randomised in a full trial?
4. Thinking about the patients that you see, are there any patient characteristics that would make you unwilling for them to be allocated to one type of implant or another by randomisation?
5. How would carrying out treatment according to study protocol affect your day-to-day working?

In the full trial, we anticipate that the surgeon would be the person who first discusses the trial with a patient before sending the patient to a research nurse to fully consent them to take part in the study (if appropriate).

1. How would you feel about discussing the trial with a patient?
   1. Would you anticipate any problems with this? *(Prompts e.g. time, patient relationship, costs?)*
   2. How confident would you feel about discussing the trial with a patient?
   3. What training do you feel you would need to enable you to have such a discussion?
2. How do you think patients might feel about being randomised to one treatment or another within a full trial?
3. What concerns do you think they might have? How might such concerns best be addressed?
4. Are there any other problems that you would foresee in recruiting patients at your hospital to take part in the trial?

**Implementation issues**

If the current feasibility study suggests we can run a full trial, the next stage of research will be a full trial. Depending on that trial’s findings, we may be recommending the use of one type of hip joint or the other.

1. How would you feel about being given guidelines recommending one approach or another?
2. How would you feel about following such guidelines?
3. What barriers do you think there might be to such recommendations being implemented in practice?
   1. How could these barriers be overcome?
4. What would facilitate the implementation of any such recommendations into practice?

**Demographic questions**

- Gender
- Year first appointed as a consultant surgeon (or no. years since appointed as a consultant)
- For how many years have you been carrying out hip replacements?
- How many such operations would you typically carry out each month?
