# Supplementary material for: Implementation of hip replacement surgery recommendations: a qualitative study of orthopaedic surgeons’ perspectives
Source: BMC Musculoskelet Disord. 2025 Dec 8;27:26. doi: 10.1186/s12891-025-09334-z (PMC12797885; doi:10.1186/s12891-025-09334-z)
Supplement: Supplementary file 3 — Additional File 3. Further analysis information. 1) Additional details on analysis process; 2) Working thematic framework (implementation categories); 3) Final thematic structure of implementation findings. [file 12891_2025_9334_MOESM3_ESM.docx]

**Additional File 3: Further analysis information**

**Analysis process:**

*Qualitative researcher information:*

AD, a research assistant with training in health psychology and research methods at MSc level, had experience of qualitative research in earlier roles. She had no previous relationship with participants and conducted all interviews. RP was an experienced researcher, held a PhD in health psychology, and supervised AD.

*Analysis:*

Interview audio-recordings were transcribed verbatim. An inductive, data-driven thematic analysis was conducted to explore and understand ‘patterns’ within the data (Braun & Clarke, 2006). We used the Framework approach to structure the analysis (Ritchie & Spencer, 1994; Spencer et al., 2014). Framework is a systematic approach with a transparency which enables other team members to follow and discuss the analysis and decisions made. An inductive rather than deductive, theoretically-driven approach was taken because we sought to focus on potential barriers to implementation of evidence-based guidance which were most salient to the surgeon participants.

Within the analysis process for the full dataset (Board et al., In submission), AD gained familiarisation with surgeon interviews by reading and re-reading transcripts, noting thoughts and ideas on transcripts. RP also read all transcripts, noting issues and ideas for each participant. AD re-read and applied codes (descriptions of apparently important ideas) to a sample of five surgeon transcripts. Codes were collated into lists, grouping codes around similar concepts. Remaining transcripts were re-read, with any new ideas coded and collated. The list of codes was organised into a ‘working thematic framework’ – a list of categories and sub-categories (below, p.2). AD completed these processes, meeting weekly with RP to discuss ideas and development of the working framework. The working framework was discussed with other research team members, ensuring that the framework contents rang true for the wider research team (including clinical team members), and gaining insights on possible interpretations.

AD applied the working framework to all transcripts (‘indexing’) using Word. AD and RP ‘charted’ the data: matrices were created in Excel mapping working framework categories onto participants with summaries of relevant data within cells, with transcript line numbers. RP led the ‘mapping and interpretation’ analysis phase, interrogating the charts to gain a deeper understanding of the dataset and developing themes; she met regularly with AD to discuss the analysis. Preliminary findings were discussed with the full research team before finalising themes. We neither shared transcripts with participants nor asked participants to provide feedback on study findings. We did not wish to increase burden of participation, especially as this practice’s value is debated (Thomas, 2017).

On completion of the inductive analysis, we related the findings to the TDF and CFIR. Mapping our findings onto theoretical constructs meant that it would be possible for our findings to be considered using labels which would be consistent with the wider literature, contributing to the wider evidence base. This mapping would also enable researchers to draw on theory and evidence when considering processes by which implementation of guidance might be optimised (Cane et al., 2012). We considered both TDF and CFIR because we did not know, a priori, which issues might be most important in the present context and so could not predict which framework would be the best fit. Mapping our findings to theory after an inductive analysis would enable us to determine which framework would be the most useful going forward – or how the frameworks might be appropriately combined for the present context.

This mapping was carried out by RP, who carefully re-read the inductive findings and considered how they might relate to the domains and constructs in each theoretical framework. The mapping was checked by VW. It was then considered how the two frameworks might usefully contribute to implementation of evidence-based guidance.

**HipHOP Qualitative Study Working Thematic Framework: Implementation categories**.

***Implementation issues*** (category 13 of the HipHOP feasibility study’s full working framework). See Board et al. (In submission) for qualitative study’s full working framework.

1. *Surgeon’s beliefs and attitudes*
   1. Difficulties changing strongly held beliefs
   2. Thoughts on strategies to change beliefs
2. *Training issues*
   1. Implementation difficult if surgeons only trained/experienced in one approach
   2. Training to facilitate implementation
3. *Impact of study findings in changing practice*
   1. Willingness to change practice based on evidence *e.g. happy to change based on evidence, too late in career to change*
   2. Strength of findings

**Final thematic structure of implementation findings:**

*Theme 1: Beliefs about best practice*

- Perceptions informing current practice
- Evidence base informing future practice
- Dissemination of trial findings and resulting recommendations

*Theme 2: In my hands*

*Theme 3: Ensuring competency*

**References:**

Board, T. N., Wylde, V., Divecha, H., Gornall, M., Jackson, R., Coffey, T., Eden, M., Dalal, G., Davies, A., Hickey, H., Spickett, H., Taylor, T., Wilson, M., & Powell, R. (In submission). Hybrid versus cemented implants for total hip replacement: A randomised feasibility study with embedded qualitative research.

Braun, V., & Clarke, V. (2006). Using thematic analysis in psychology. *Qualitative Research in Psychology, 3*, 77-101.

Cane, J., O'Connor, D., & Michie, S. (2012). Validation of the Theoretical Domains Framework for use in behaviour change and implementation research. *Implementation Science, 7*, 37. <https://doi.org/10.1186/1748-5908-7-37>

Ritchie, J., & Spencer, L. (1994). Qualitative data analysis for applied policy research. In A. Bryman & R. G. Burgess (Eds.), *Analysing Qualitative Data* (pp. 173-194). Routledge.

Spencer, L., Ritchie, J., Ormston, R., O'Connor, W., & Barnard, M. (2014). Analysis: principles and processes. In J. Ritchie, J. Lewis, C. McNaughton Nicholls, & R. Ormston (Eds.), *Qualitative Research Practice: A Guide for Social Science Students & Researchers* (pp. 267-293). SAGE Publications Ltd.

Thomas, D. R. (2017). Feedback from research participants: Are member checks useful in qualitative research? *Qualitative Research in Psychology, 14*, 23-41. <https://doi.org/10.1080/14780887.2016.1219435>
