# Supplementary material for: Implementation of hip replacement surgery recommendations: a qualitative study of orthopaedic surgeons’ perspectives
Source: BMC Musculoskelet Disord. 2025 Dec 8;27:26. doi: 10.1186/s12891-025-09334-z (PMC12797885; doi:10.1186/s12891-025-09334-z)
Supplement: Supplementary file 4 — Additional File 4. Mapping findings to theory-based frameworks. Document relating study findings to the theoretical domains identified within the Theoretical Domains Framework (Cane et al, 2012; Michie et al, 2005) and the constructs identified within the Consolidated Framework for Implementation Research (Damschroder et al, 2009). [file 12891_2025_9334_MOESM4_ESM.docx]

**Additional File 4: Mapping findings to theory-based frameworks**

**Relating findings to the Theoretical Domains Framework (TDF) (Cane et al., 2012; Michie et al., 2005)**

In this section, we reflect on the present study’s findings and relate them to the theoretical domains identified within the TDF. We consider those TDF domains which seem most clearly relevant to the present study’s context. See Figure AF4.1 summarising mapping of key study findings onto TDF domains.

***Knowledge***

The ‘Knowledge’ domain encompasses both ‘Knowledge: An awareness of the existence of something’ and also ‘Procedural knowledge: Knowing how to do something’ (Additional File 1 in Cane et al., 2012 ).

Knowledge appears to be pertinent to our findings in terms of the perceived necessity of trial findings and resulting recommendations being disseminated effectively to facilitate implementation, with trustworthy sources such as scientific journals and professional organisations seeming valued. Having evidence of good quality seemed of importance to the present study’s participants, with a sense that it’s not enough for individuals to know about trial findings or recommendations, but that the knowledge that they are accessing should be trustworthy. Participants seemed to balance the need for knowledge of the evidence base with knowledge of their own, personal results – what seems to be working for them, ‘in their hands’.

Procedural knowledge - knowing how to do something - would appear to be of importance in the context of potentially changing surgical approach, but participants’ concerns seemed more focussed on the Skills’ domain – ‘an ability or proficiency acquired through practice’ (Cane et al., 2012, p13).

***Skills***

The ‘Skills’ domain includes the constructs of Skills development, Competence and Practice, all of which seemed to be important within our findings. Surgeons’ skill level with a recommended implant seemed crucial to the likelihood that they would be willing to use it, with implementation seeming more straightforward for surgeons who were already skilled in the recommended approach. Concerns were raised where surgeons might lack competence, or where changing practice would mean a learning curve, with poorer outcomes for patients receiving surgery earlier in the change process.

How surgeons could develop skills was considered by participants. Training needs both for current and new surgeons were discussed.

***Beliefs about capabilities***

This domain includes the constructs Perceived competence, Self-efficacy and Professional confidence, all of which relate to an individual’s perceptions of their ability to effectively carry out a specific task. Self-efficacy beliefs are proposed to influence the likelihood of attempting, and persevering with, a behaviour (Bandura, 1977). Confidence in ability to use a specified implant featured in our findings, but underlying this seemed to be a concern about surgeons’ skills, as discussed under ‘Skills’. According to Bandura, ‘performance accomplishments’ influence self-efficacy, with successful performance increasing self-efficacy (Bandura, 1977). Supporting surgeons with appropriate training and opportunities to gain experience of performing the recommended surgery with success would seem likely, therefore, to ensure surgeons achieve the needed skill level, and gain confidence in their ability to successfully use the recommended implant.

***Beliefs about consequences***

Surgeons’ beliefs about consequences of following guidelines, and potentially changing practice, seemed to both be of high importance and complex. Participants appeared to require good quality evidence to indicate that there would be clear potential benefits in outcomes to consider changing practice. It was acknowledged that surgeons’ perceptions of best practice might not always be in line with evidence, and dissemination of findings and recommendations by respected routes, such as reputable scientific journals and professional organisations, seemed considered to be likely to influence beliefs about the value of changing practice (see ‘Knowledge’ above).

However, surgeons did not just look at evidence gained from looking at group-level outcomes within clinical trials or registry databases. They also monitored their own personal results, recognising that their own individual skill with implants could influence surgical outcomes, and appearing reluctant to consider changing practice where they were not confident that the outcomes would be as good for them as individuals, particularly during a learning curve phase. It therefore seems possible that a surgeon might concurrently hold beliefs that the consequences of using an implant would be positive in hip surgery generally but negative for their own hip replacement practice.

Surgeons also seemed to consider patients at an individual level, such that whilst the recommended implant might be believed to provide the best results for patients in general, surgeons sought to retain autonomy to use their clinical judgement to decide what approach would be best for an individual patient, with their individual characteristics.

***Environmental context and resources***

This domain seems particularly pertinent to the theme ‘ensuring competency’, where a need for training to support both changing practice in current surgeons, and also appropriate training for new surgeons was identified. In both contexts, resources would be needed to support training, and these are likely to be needed at organisational and higher levels – e.g. national programmes to provide or amend training.

***Social influences***

Surgeons seemed to be influenced by the thoughts and behaviours of their peers. It has been noted above that guidelines being supported by professional organisations was viewed as likely to support implementation. Such organisations might be expected to indicate accepted, prevailing views within the discipline – i.e. the views of other, influential surgeons. A perceived pressure to practice in line with colleagues was also mentioned, such that carrying out a different practice to others seemed to carry potential risk.

| **HipHOP findings summarised by theme** |  | **TDF domains** |
| --- | --- | --- |
| 1. **Beliefs about best practice**   Perceptions informing current practice   - Belief that change → better outcomes - Beliefs about treatments best fitting with individual patient characteristics - Beliefs about evidence (e.g. NJR data; studies)   Evidence base informing future practice   - Importance of good quality evidence with clear indication of best practice - Wariness of poorly conducted trials; scepticism of trial’s ability to give conclusive answers regarding clinical outcomes. - Perceptions of best practice not always in line with evidence.   Dissemination of trial findings and resulting recommendations   - Reputable scientific journals; respected organisations - Change at group/team level – having similar practice to peers - Desire to retain autonomy – use clinical judgement for individual cases  1. **In my hands**  - Awareness of personal results – evidence of what is working in own practice - Skill level with specific procedure; skill related individual’s training and experience - Concern about changing practice where learning curve could impact results - Implementation more straightforward where surgeons are already skilled in recommended procedure.  1. **Ensuring competency**  - Need for training to support change in surgical practice - Desire for established surgeons to retrain unclear - Potential to train new surgeons in procedure supported by evidence – would need support from bodies specifying training requirements. |  | 1. ***Knowledge*** 2. ***Skills*** 3. Social/professional role and identity 4. ***Beliefs about capabilities*** 5. Optimism 6. ***Beliefs about consequences*** 7. Reinforcement 8. Intentions 9. Goals 10. Memory, attention and decision processes 11. ***Environmental context and resources*** 12. ***Social influences*** 13. Emotion 14. Behavioural regulation |

Notes: NJR = National Joint Registry; TDF = Theoretical Domains Framework.

**Figure AF4.1: Mapping summary of findings to constructs in Theoretical Domains Framework (Cane et al., 2012)**

**Relating findings to constructs in the Consolidated Framework for Implementation Research (Damschroder et al., 2009)**

In this section, we reflect on the present study’s findings and relate them to constructs identified by Damschroder et al.’s CFIR. We consider those CFIR constructs which seem most clearly relevant to the present study’s context, drawing on Damschroder et al.’s descriptions of these constructs (Damschroder et al., 2009). See Figure AF4.2 summarising mapping of key study findings onto CFIR constructs.

***Evidence strength and quality***

This CFIR construct identifies that the views of stakeholders respecting evidence quality may impact their beliefs about an intervention’s beliefs on outcomes. Participants’ perceptions of evidence appeared to be important in the present findings, with participants considering various sources of evidence. Traditional sources of population-level evidence, such as research studies and national registry data, were referred to by participants. At the same time, some participants suggested that surgeons’ perceptions of best practice were not necessarily in line with the evidence base. Further, it seemed that surgeons also consider their own experience and personal data as a source of evidence: individuals appeared to reflect on their own practice in terms of identifying what went well for them, and monitored data related to their personal practice.

***Relative advantage.***

Relative advantage – perception of the intervention as opposed to another treatment – appeared to be of relevance throughout the present study’s findings. Participants were considering following evidence-based guidance to indicate which type of implant to use, as opposed to using an implant in line with their personal clinical judgement. There was, therefore, a sense that individuals needed to be persuaded that following guidance would lead to better results than they were currently achieving – results which could already be perceived to be good.

***Complexity***

The intervention could be considered ‘complex’ in that implementing the intervention requires specific, high-level skills of trained surgeons.

***Cost***

Cost of the intervention as such did not seem to be a barrier or facilitator to implementation within the present findings. However, it seemed that cost-effectiveness was valued such that if there was no clinical difference between implant types, the benefit to health services of using the cheaper implant was noted.

***Patient needs and resources***

The desire to meet patient needs featured in the present study findings, with participants highlighting the perceived importance of using implants which suited individual patients’ characteristics. Surgeon participants also appeared concerned about making changes to surgical practice whereby a learning curve could negatively impact surgery outcomes. When discussing the HipHOP trial with patient participants in the feasibility study, in general, it seemed that patients were happy to receive either implant type, if it was recommended by a trusted surgeon (Board et al., In submission). It therefore seems important to ensure that surgeons are happy that the recommended implant would meet their patients’ needs.

***Peer pressure***

Peer pressure seemed relevant to the present study findings in two main respects. Dissemination of trial findings and practice recommendations from respected professional organisations seemed influential, and carrying out different practice to peers appeared to be seen as risky. It would therefore seem likely to be reassuring if a change in surgical practice was implemented by others, in similar ways, rather than focussing on change in individual surgeons, and for that change to be recommended by respected leaders and organisations in the field.

***External policies and incentives***

Two areas in which external policies or strategies seemed relevant to the present findings were a) the development of guidelines, with guidelines disseminated by respected organisations such as professional bodies seeming to have potential influence, and b) in addressing training needs, with the suggestion that fundamental changes in training would require support from bodies that develop medical training standards and programmes.

***Networks and communications***

Our findings identified an apparent desire to conduct surgical practice in line with surgical peers (see ‘Peer pressure’). Involving effective use of social and communication networks within surgical communities could therefore facilitate practice change.

***Implementation climate***

This is a broad construct within the CFIR containing six sub-constructs, some of which seemed relevant to the present study context. *‘Tension for change’* appeared to be of importance, with participants indicating that they would wish to be convinced that changing practice would lead to improved outcomes. *‘Compatibility’* of the intervention with professional values may also influence implementation, with participant responses suggesting that having evidence-based guidelines to follow was perceived to be of value, but with participants also indicating a desire to retain the autonomy to use their clinical judgement for individual cases. A blanket requirement to follow guidance over implant choice might not be seen as compatible with the freedom to use clinical judgement. *‘Learning climate’* seems to be a particularly relevant sub-construct: the need for surgeons to have the appropriate skill level to use an implant type was a concern for participants.

***Knowledge and beliefs about the intervention***

Beliefs about which implants would give the best outcome seemed central when considering implementation; this construct directly maps onto our first theme ‘Beliefs about best practice’. Evidence from a national registry and trials was recognised as valuable, but evidence from participants’ own experience, and their own personal records, was also an important source of information appearing to influence beliefs around best practice.

***Self-efficacy***

See ‘Beliefs about capabilities’ (TDF section) for discussion of issues around self-efficacy beliefs.

***Other personal attributes***

This construct includes ‘competence’. Competency in one’s own practice, and ensuring competency across the discipline, were discussed within interviews.

| **HipHOP findings summarised by theme** |  | **CFIR constructs (Damschroder et al 2009)** |
| --- | --- | --- |
| 1. **Beliefs about best practice**   Perceptions informing current practice   - Belief that change → better outcomes - Beliefs about treatments best fitting with individual patient characteristics - Beliefs about evidence (e.g. NJR data; studies)   Evidence base informing future practice   - Importance of good quality evidence with clear indication of best practice - Wariness of poorly conducted trials; scepticism of trial’s ability to give conclusive answers regarding clinical outcomes. - Perceptions of best practice not always in line with evidence.   Dissemination of trial findings and resulting recommendations   - Reputable scientific journals; respected organisations - Change at group/team level – having similar practice to peers - Desire to retain autonomy – use clinical judgement for individual cases |  | 1. Intervention characteristics 2. Intervention source 3. ***Evidence strength and quality*** 4. ***Relative advantage*** 5. Adaptability 6. Trialability 7. ***Complexity*** 8. Design quality and packaging 9. ***Cost*** |
|  |  | **2.** Outer setting  ***A. Patient needs and resources***  B. Cosmopolitanism  ***C. Peer pressure***  ***D. External policy and incentives*** |
|  |  | **3.** Inner setting  A. Structural characteristics  ***B. Networks & communications***  C. Culture  ***D. Implementation climate***  E. Readiness for implementation |
|  |  | **4.** Characteristics of individuals  A. Knowledge and beliefs about the intervention  ***B. Self-efficacy***  C. Individual stage of change  D. Individual identification with organisation  ***E. Other personal attributes (includes ‘competence’)*** |
| 1. **In my hands**  - Awareness of personal results – evidence of what is working in own practice - Skill level with specific procedure; skill related individual’s training and experience - Concern about changing practice where learning curve could impact results - Implementation more straightforward where surgeons are already skilled in recommended procedure. |  |  |
|  |  | 5. Process  A. Planning  B. Engaging  C. Executing  D. Reflecting and evaluating |
| 1. **Ensuring competency**  - Need for training to support change in surgical practice - Desire for established surgeons to retrain unclear - Potential to train new surgeons in procedure supported by evidence – would need support from bodies specifying training requirements. |  |  |

Notes: NJR = National Joint Registry; CFIR = Consolidated Framework for Implementation Research.

**Figure AF4.2: Mapping summary of findings to constructs in the Consolidated Framework for Implementation Research (Damschroder et al., 2009)**

**References:**

Bandura, A. (1977). Self-efficacy: Toward a unifying theory of behavioral change. *Psychological Review, 84*(2), 191-215. <https://doi.org/10.1037/0033-295X.84.2.191>

Board, T. N., Wylde, V., Divecha, H., Gornall, M., Jackson, R., Coffey, T., Eden, M., Dalal, G., Davies, A., Hickey, H., Spickett, H., Taylor, T., Wilson, M., & Powell, R. (In submission). Hybrid versus cemented implants for total hip replacement: A randomised feasibility study with embedded qualitative research.

Cane, J., O'Connor, D., & Michie, S. (2012). Validation of the Theoretical Domains Framework for use in behaviour change and implementation research. *Implementation Science, 7*, Article number 37. <https://doi.org/10.1186/1748-5908-7-37>

Damschroder, L. J., Aron, D. C., Keith, R. E., Kirsh, s. R., Alexander, J. A., & Lowery, J. C. (2009). Fostering implementation of health services research findings into practice: A consolidated framework for advancing implementation science. *Implementation Science, 4*, 50, Article number 50. <https://doi.org/10.1186/1748-5908-4-50>

Michie, S., Johnston, M., Abraham, C., Lawton, R., Parker, D., Walker, A., & on behalf of the "Psychological Theory' Group. (2005). Making psychological theory useful for implementing evidence based practice: A consensus approach. *Quality & Safety in Health Care, 14*, 26-33. <https://doi.org/10.1136/qshc.2004.011155>
